# Force Profile Characteristics of Gravitational and Pneumatic Resistances in Pull and Push Exercises

**DOI:** 10.3390/sports13080239

**Published:** 2025-07-22

**Authors:** Manuel Barba-Ruiz, Juan Ramón Heredia-Elvar, Adrián Martín-Castellanos, Javier Iglesias-García, Francisco Hermosilla-Perona

**Affiliations:** 1 AExPH, Facultad de Ciencias Biomédicas y de la Salud, Universidad Alfonso X El Sabio, 28691 Madrid, Spain; mruizbar@uax.es (M.B.-R.); jelvaher@uax.es (J.R.H.-E.); adrimaca@uax.es (A.M.-C.); javiigga@uax.es (J.I.-G.); 2 Facultad de Ciencias de la Vida y la Naturaleza, Universidad Nebrija, 28248 Madrid, Spain

**Keywords:** training, strength, biomechanics, inertial

## Abstract

Introduction: Strength training, essential for health and performance, often uses free weights for greater stabilization demands and pulleys for easier load adjustment and progression. Methods: The aim of the study was to analyze the differences in force application using gravitational and pneumatic resistances. Twenty experienced subjects participated in the study (age: 21.9 ± 3.8 years; body mass: 76.3 ± 9.4 kg; height: 177.4 ± 7.5 cm), performing four exercises with each type of resistance: bench press, lat pulldown, chest fly, and single-arm row. The participants performed 8 repetitions per exercise. Peak and mean force were measured with a 100 Hz load cell (SUIFF S2 Pro) during the concentric phase of the lifts. Differences between resistance types were analyzed using one-way ANOVA and paired t-tests. Results: Peak force was higher with gravitational resistance across all exercises (*p* < 0.001; d = 2.1–4.7). Average force with gravitational resistance was also higher in the bench press and lat pulldown (*p* < 0.05; d = 0.7–1.4), but not in the chest fly or single-arm row. Conclusions: Gravitational resistance may better enhance peak strength, while pneumatic resistance supports consistent force and neuromuscular control. These results allow us to select the resistance type based on specific mechanical characteristics.

## 1. Introduction

Strength, a fundamental physical capacity, refers to the maximum force that a muscle or muscle group can produce at a given velocity [1]. Strength training is a crucial element in fostering health and physical performance, benefiting both elite athletes and individuals seeking to enhance their fitness and well-being [2]. Traditionally, strength training has been executed using free weights or machines with guided weights on pulley systems. Free training, involving implements like dumbbells, barbells, and disks, eschews machines or pulley systems. In contrast to pulley training, free training demands heightened muscle stabilization and coordination, requiring muscles to collaboratively control and move the load. The natural range of motion in free weight training seems to allow for increased engagement of stabilizer muscles and greater functional transfer to daily activities or specific sports compared to machine or pulley resistance [3]. Pulley training provides the benefit of easily adjusting the load, enabling progressive resistance increases, and may also impose greater stability demands compared to free weight exercises. For instance, pressing movements such as the standing cable press impose greater demands on trunk and core stability compared to the bench press [4].

Thus, in dynamic exercises involving free-weight resistance, it is necessary to generate peak forces approximately twice the load weight to attain the heightened acceleration characteristics of lighter lifts [5,6]. In this context, the lifter must first apply sufficient force to overcome the gravitational pull of the load (mass × gravity). This typically involves an initial isometric phase lasting approximately 40–100 ms before movement begins [7]. Once this threshold is surpassed, a rapid application of force is required to accelerate the load, often resulting in peak force values that substantially exceed the load itself. However, this initial burst of force also generates momentum, which causes the load to continue moving even as muscular force begins to decline. As a result, force output can drop below the load’s weight later in the range of motion, as the lifter must decelerate the movement to bring it to a controlled stop [8,9]. This negative acceleration phase within the concentric portion of the lift can be considerable. For example, Elliott et al. [10] reported that during a bench press with 81% of 1RM, the force applied by the lifter fell below the resistance load for approximately two-thirds of the concentric range of motion. These findings underscore the complex mechanical characteristics inherent in traditional resistance modes and are supported by a substantial body of literature exploring the kinetics of various resistance modalities, including elastic, chain, pneumatic, and accommodating systems [10,11]. In this context, inertial load contractions effectively generate peak forces near the initiation of the concentric phase of the lift [12]. However, force later in the range of motion decreases due to the force–velocity relationship of muscle and the physics of a zero-velocity endpoint. This decline in force late in the range of motion is reduced with thrown/projected weight systems [11].

One alternative developed to address the limitations of inertial load involves the use of pneumatic resistance. Pneumatic loading entails creating variable resistance by compressing air in a cylinder [13]. When employing this technique (pneumatic resistance), the only inertia to overcome is associated with the mass of the mobilized body segments, bar, and cable, thereby limiting the amount of force required to initiate the movement [14,15]. Consequently, pneumatic resistance has been shown to reduce force variability throughout the concentric phase due to minimal inertial effects, promoting a more uniform application of force compared to traditional weight-loaded systems. Moreover, when load magnitude is equivalent, pneumatic resistance allows for higher movement velocities, particularly in the late phase of concentric action, due to the reduced need for deceleration forces [15].

When analyzing the impact of different resistances on strength outcomes, it is essential to consider not only the type of resistance (e.g., gravitational or pneumatic) but also the specific exercises and body positions adopted. The selection of exercises and their corresponding body positions significantly influence muscle recruitment patterns, joint loading, and force application dynamics [16,17]. These variations affect stabilization requirements, the direction of applied resistance, and overall force output [18]. Therefore, assessing a range of exercises performed in distinct body positions allows for a better understanding of the influence of resistance type on force production, thereby enhancing the ecological validity of the findings in the context of applied strength training.

Hence, the aim of this study was to analyze and compare the variations in force application between gravitational and pneumatic resistance in different exercises. The hypothesis of this study proposes that gravitational resistance is expected to generate higher peak force outputs and greater variability in force application compared to pneumatic resistance.

## 2. Materials and Methods

### 2.1. Experimental Approach to the Problem

This study used a within-subject, counterbalanced, and repeated-measures design. The participants completed the exercises with both gravitational and pneumatic resistance, incorporating 2 min rest periods between each exercise. The training volume was standardized across both resistance types, with participants performing 8 repetitions per exercise under each condition, using identical loads for the same exercise in both gravitational and pneumatic resistance.

To ensure ecological validity, the participants maintained their normal unsupervised diet and training, which were not recorded. The participants, however, were asked to avoid (a) alcohol for 48 h, (b) exercise for 24 h, and (c) caffeine for 8 h before each visit.

### 2.2. Participants

Twenty male participants with at least two years of strength training experience (age: 21.9 ± 3.8 years; body mass: 76.3 ± 9.4 kg; height: 177.4 ± 7.5 cm) participated in this study. To be eligible, participants had to meet specific inclusion criteria: (I) age ranging from more than 18 years to less than 30 years, (II) a minimum of 2 years of experience in strength training, and (III) familiarity with the exercises conducted in the study. Additionally, participants were required to be free of musculoskeletal injuries for a period of at least 6 months before the study commencement. A priori power analysis using a paired *t*-test (α = 0.05) and large effect size (based on previous analysis) indicated that a sample size of 20 participants would yield a statistical power of 85%.

Participants were recruited from local fitness centers and university sports programs. Detailed explanations of the experimental procedures were provided to the participants, who were thoroughly briefed on the associated risks. Prior to their involvement in the study, the participants provided written consent. This study was conducted in compliance with the guidelines provided by the Institutional Ethics Review Board at the local level. The study protocol received formal approval from the same Institutional Ethics Review Board. Furthermore, all participants provided written informed consent, aligning with the principles set forth in the Declaration of Helsinki.

### 2.3. Procedure

The order of resistance conditions (gravitational and pneumatic) was randomized for each participant to control for sequence effects. To prevent any bias, the participants performed the exercises in the same order between resistance types.

Due to the difference between the dual gravity pulley (BH L370 Dual Adjustable Pulley, Vitoria Gasteiz, Basque Country, Spain) and the pneumatic pulley (KEISER, Fresno, CA, USA), measurements were taken for the participants’ positioning distances and the angles of these pulleys. This was done to minimize discrepancies in the angular orientation of force directions. A manual universal plastic goniometer (Baseline^®^ 360-degree, Fabrication Enterprises Inc., Elmsford, NY, USA) was used to assess initial joint angles. This tool allows for accurate measurement of range of motion through its full-circle scale and two adjustable arms. This procedure was carried out during a familiarization session in which the participants performed all the exercises using 50% of the load later prescribed in the experimental session. A 24 h gap was maintained between these two sessions.

**Figure 1:**
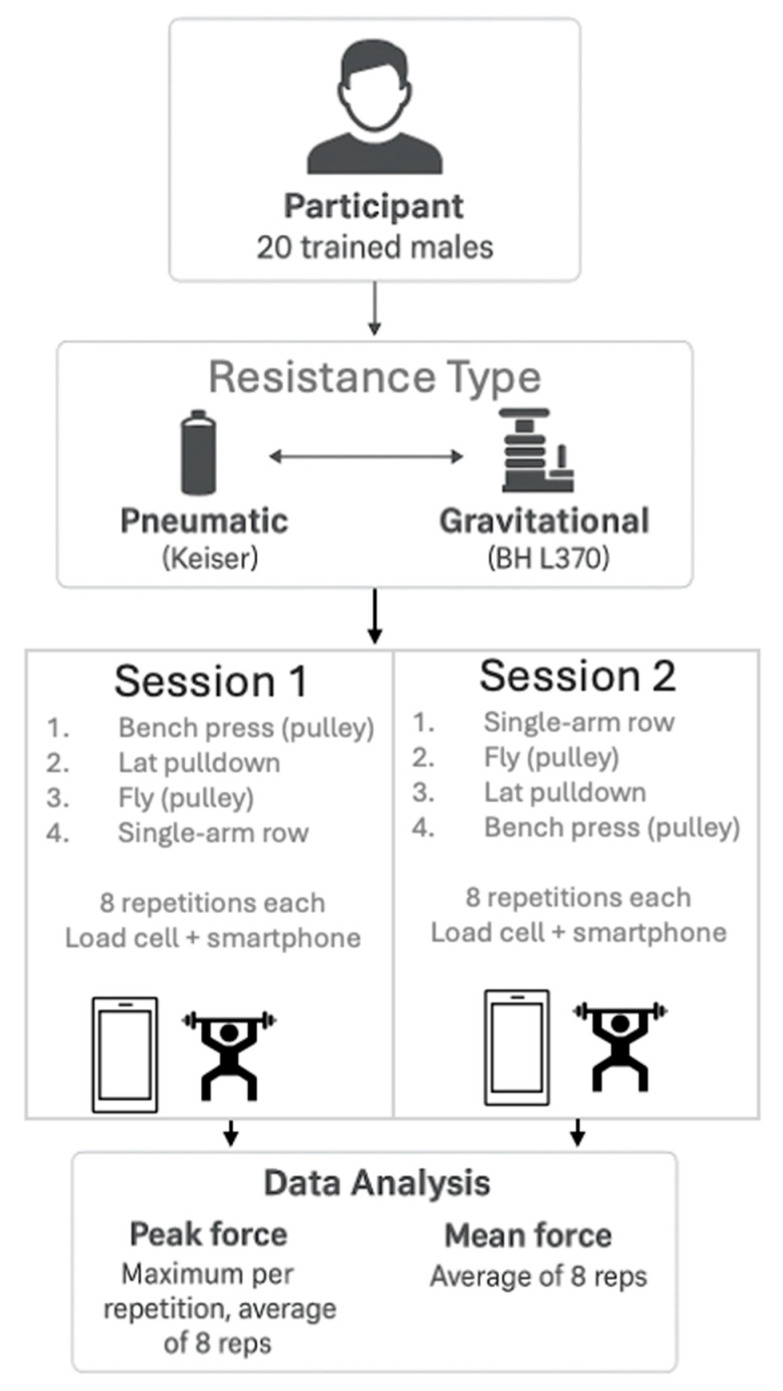
Experimental protocol and instruments.

Each participant completed a total of eight exercises, with four exercises on the gravity pulley and the same four exercises on the pneumatic pulley. The selection of the exercises and their corresponding loads was based on a combination of prior literature and the participants’ physical characteristics. Specifically, exercises were chosen for their relevance to upper-body strength development and their feasibility within both gravity-based and pneumatic pulley systems [19,20]. The exercises included bench press using a pulley system (total load: 30 kg; 15 kg per arm), lat pulldown (total load: 12.5 kg; 6.25 kg per arm), dumbbell fly with pulley resistance (total load: 17.5 kg; 8.75 kg per arm), and single-arm row (load: 11.25 kg), performed exactly in that order. A 2 min rest interval was prescribed between the exercises and sets. In each exercise, the participant carried out 8 repetitions. Also, the participants were instructed to perform all the repetitions as fast and forcefully possible (applying the maximal voluntary force in each repetition). The load was standardized across all participants to enable comparisons between individuals and resistance (Figure 1).

**Figure 2:**
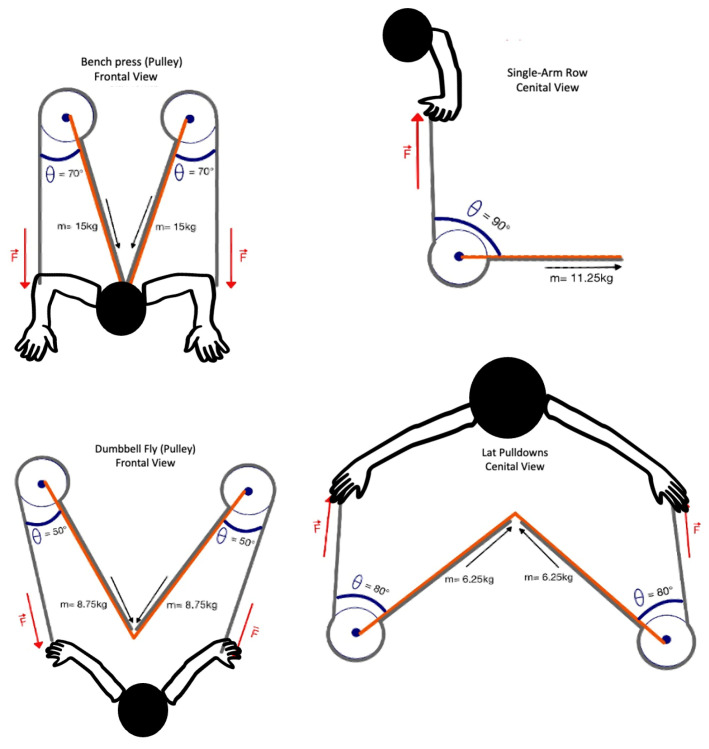
Angular positions and resistance of the exercises performed.

All the exercises were carried out with the same initial elbow and shoulder angles in order to maintain the same characteristics between gravitational and pneumatic resistance. In this sense, the participants were placed at marked positions previously analyzed with a goniometer (Figure 2).

### 2.4. Data Analysis

The assessment of peak and average force was carried out using a load cell (SUIFF S2 Pro, Barcelona, Spain) connected to a smartphone (Samsung Galaxy S10, Samsung electronics Co., Suwon, Republic of Korea) with a sampling rate of 100 Hz. The load cell was linked to the resistance using its own carabiners on both the traditional and pneumatic pulley systems.

The load cell, positioned between the grip and the wire connected to the Keiser and the weight plates in the dominant arm, gathered all strength-related variables. It recorded each repetition in every exercise, capturing both the peak and the average force. The peak force was defined as the maximum instantaneous force recorded during each individual repetition. The values from all 8 repetitions were then averaged to obtain peak force for that exercise and resistance condition. The mean force was calculated as the average force over the duration of each repetition. As with peak force, mean values from all 8 repetitions were averaged to obtain a single representative mean force value per exercise and condition. The smartphone application facilitated the provision and exportation of all the collected data (Suiff Pro App, Estel Grup Innova S.L., Madrid, Spain).

### 2.5. Statistical Analyses

Mean ± standard deviation (SD) was obtained for the descriptive analysis of the study variables. The normality of the data distribution was verified and confirmed using the Shapiro–Wilks test.

One-factor analysis of variance (ANOVA) was conducted to assess variations in both peak and mean force in each exercise between conditions (2 × 2) (gravitational and pneumatic resistance). Each dependent variable (peak force and mean force) was analyzed separately to assess the effect of the resistance type. Additionally, eight paired t-tests were employed to examine the differences between the resistance types within each exercise. For graph purposes, a linear regression line with 95% confidence intervals was included to visually represent the relationship between resistance condition and both peak and mean force.

All analyses were performed using SPSS v. 24.0 statistical software for Mac OS (IBM SPSS Statistics, New York, NY, USA) and GraphPad Prism 8 software (GraphPad Software Inc., La Jolla, CA, USA). In addition, Jamovi software (v. 1.1.9.0) was used for the descriptive analysis of the study variables. The significance level was set at *p* < 0.05. The effect size was calculated using Cohen’s d (d). Cohen’s effect sizes were classified as small (d = 0.20–0.49), medium (d = 0.50–0.79), and large (d ≥ 0.8) [21].

## 3. Results

Descriptive statistics for the different exercises performed under both gravitational and pneumatic resistance conditions are presented in Table 1.

**Figure 3:**
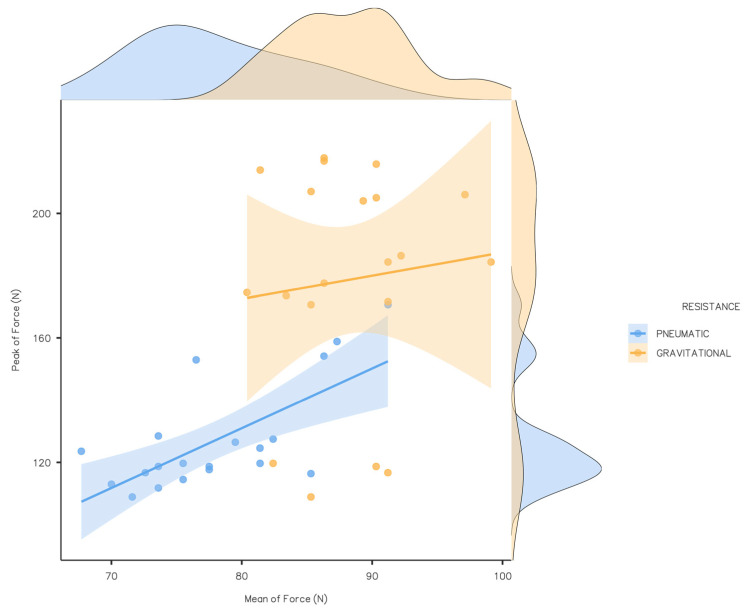
Mean of force and peak force descriptive data according to each resistance in all the exercises performed. Note: Lines represent a linear regression with shaded areas indicating 95% confidence intervals.

Examining the distribution of peak force data (Figure 3), it is evident that in pneumatic resistance, values are concentrated around 120 N, with lower variations. However, gravitational resistance presents a very different pattern, with higher variations and less consistent application of force across the repetitions. Also, the average force in pneumatic resistance exhibits a more concentrated grouping around 75 N, while in gravitational resistance, a smaller but discernible grouping is observed, albeit larger than that on the Y-axis (peak force).

**Figure 4:**
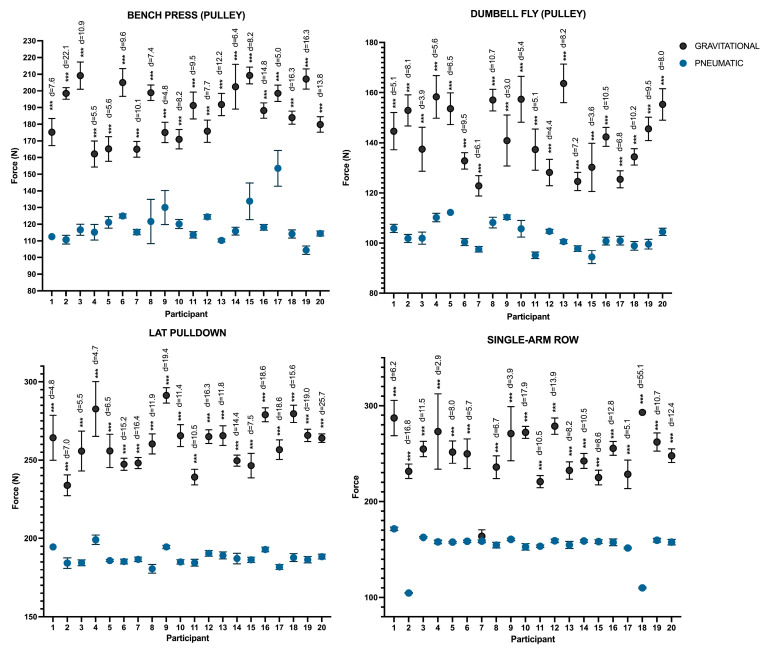
Differences in peak force between each resistance according to the participants. Note: *** *p* < 0.001. Note: Mean and SD are presented in the graph.

The findings regarding the maximal force applied during all repetitions by each participant (Figure 4) revealed significant differences between the resistance types, with large effect sizes across all participants and exercises (*p* < 0.001; d:2.9–55.1). The majority of the participants in the rowing exercise reported consistently higher levels of gravitational resistance. Additionally, while some participants showed the opposite pattern, the majority exhibited greater force variability under gravitational resistance compared to pneumatic resistance.

**Figure 5:**
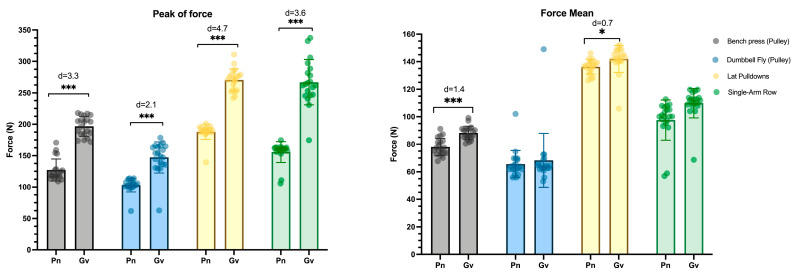
Differences in peak force and mean force in the different exercises performed under each resistance. Note: Pn: pneumatic; Gv: gravitational; * *p* < 0.05; *** *p* < 0.001.

Regarding the peak force, noteworthy distinctions (*p* < 0.001; d: 2.1–4.7) are apparent between pneumatic and gravitational resistance, consistently favoring higher forces in gravitational resistance across all exercises (Figure 5). Also, the average force shows significant differences in the press with a large effect size (*p* < 0.001: d = 1.43) and in pull-up exercises with a moderate effect size (*p* < 0.05: d = 0.7). However, in the opening and rowing exercises, no notable differences were observed between the two resistances.

## 4. Discussion

The main results of the study indicate that gravitational resistance consistently showed higher peak forces than pneumatic resistance, with predominantly large effect sizes observed both across exercises and within individual participants. This result could be attributed to the acceleration-induced increase in inertial forces in gravitational resistance, resulting in reduced applied force [22]. These findings align with a prior study in which pneumatic and free weight loads exhibited similar mean force values. However, when it comes to peak force, distinctions become more pronounced, consistently indicating higher values with gravitational loads [15].

The force application pattern reveals notable distinctions between the resistances. Gravitational resistance exhibits, in general, higher relative variability in force, as indicated by greater coefficients of variation (CVs), while pneumatic resistance demonstrates a more consistent force output across repetitions. These findings align with previous studies indicating that pneumatic resistance maintains a relatively constant force application across the range of motion, mitigating the impact of momentum in exercises with older women [23]. In terms of mechanical power, a similar study highlighted distinct differences between pneumatic and free-weight loads. It was found that pneumatic loads produce lower maximum power values with less pronounced peaks (greater linearity) throughout repetitions [19]. However, these differences are less pronounced compared to the force application patterns observed in our study, which show greater variability across repetitions. This is likely due to the interplay between force and velocity, as pneumatic loads tend to generate lower peak force values while maintaining similar velocities during the entire range of movement [15].

Taking into account the differences in force and velocity, it can be hypothesized that both resistance types can induce different muscle performance adaptations. Pneumatic resistance provides nearly constant resistance throughout the entire range of motion [23], whereas gravitational resistance varies based on body position, potentially impacting muscular work and physical adaptations [24]. In addition, using pneumatics will eliminate the impact of momentum, offering a chance to establish a coordination strategy that can be more effective in sustaining force production across the entire range of motion. This characteristic could be beneficial for optimizing neuromuscular coordination during high-velocity training, although not necessarily enhancing the maximal rate of force development (RFD) across the entire range [15].

Another factor to consider is that the precise adjustment of the load in pneumatic resistance allows for greater control of exercise intensity and training variety. This contrasts with the larger increments and potential limitations in intensity adaptation and variety associated with gravitational resistance [19]. Movement patterns during exercise may differ between the two resistance types due to variations in the way resistance is applied. For instance, pneumatic resistance might offer greater resistance in the entire range of movement during the concentric phase, while gravitational resistance may provide more resistance during the transition between concentric and eccentric phases and the first instants of concentric movement [25]. In this sense, the differences in the force profile during the concentric phase can provide several practical applications related to designing resistance training strategies for specific goals, such as improving maximal strength or power in athletes, enhancing neuromuscular control in rehabilitation settings, or promoting safe strength development in clinical populations [26]

Finally, combining both resistances could be a possible strategy for providing a varied strength-oriented stimulus compared to using either resistance alone, as long as the potential drawbacks of one resistance type could be offset by the benefits of the other [27]. Additional research could involve designing experiments to analyze the long-term impacts of various training methods, either by combining or comparing these two types of resistance.

From a practical standpoint, these force and velocity differences may lead to distinct physiological adaptations. Pneumatic resistance offers a constant load regardless of body position, potentially enhancing muscular control and consistency in work output. It also reduces the influence of momentum, which could benefit the development of the rate of force production across full movement. In contrast, gravitational resistance varies with position and may increase muscular demand during the transition and early concentric phases, possibly supporting gains in peak strength. Furthermore, movement patterns may shift depending on the resistance type, reinforcing the need to tailor resistance selection to the desired adaptation—whether for power, strength, or neuromuscular control.

Among the limitations of this study, it should be noted that although force was accurately measured, velocity data were not recorded, which prevented a comprehensive analysis of the force–velocity relationship. Additionally, the sample consisted exclusively of young male participants with prior strength training experience, limiting the generalizability of the findings to other populations, such as females, older adults, or individuals without training experience. Also, timing and speed of repetitions were not strictly controlled, and differences in eccentric phases may have influenced the force recorded. These factors should be considered when interpreting the results.

## 5. Conclusions

In summary, the differences between inertial and pneumatic resistance are associated with disparities in peak force and mean force during the concentric phase in all the push and pull exercises studied. In pulley systems, gravitational resistance consistently produces higher force peaks in comparison to pneumatic resistance; however, these differences may also be mediated by the specific characteristics of each exercise. Conversely, pneumatic resistance exhibits a more consistent average force across all exercises. Therefore, the choice between these resistance types should consider specific training objectives and the distinct force application characteristics highlighted in this study.

## Figures and Tables

**Table 1:** Descriptive data of the variables analyzed.

	n = 20	Shapiro–Wilks (*p*-Value)	Mean	Standard Deviation (SD)	Coefficient of Variation	Min	Max
Peak of Force (N)							
Gravitational	Bench press (pulley)	0.915	196.6	15.78	0.08	171.7	217.8
	Dumbbell fly (pulley)	0.809	147.1	24.75	0.16	62.8	178.5
	Lat pulldown	0.967	270.5	17.53	0.06	241.3	311.0
	Single-arm row	0.943	266.9	36.04	0.13	174.6	337.5
Pneumatic	Bench press (pulley)	0.793	127.2	17.50	0.13	108.9	170.7
	Dumbbell fly (pulley)	0.672	103.0	10.91	0.10	61.8	113.8
	Lat pulldown	0.554	188.0	12.16	0.06	139.3	201.1
	Single-arm row	0.573	155.7	16.70	0.10	105.9	174.6
Average force (N)							
Gravitational	Bench press (pulley)	0.952	88.2	4.89	0.05	80.4	99.1
	Dumbbell fly (pulley)	0.451	68.3	19.58	0.28	53.0	149.1
	Lat pulldown	0.702	142.0	9.84	0.06	105.9	152.1
	Single-arm row	0.640	109.8	10.68	0.09	68.7	119.7
Pneumatic	Bench press (pulley)	0.969	78.0	6.24	0.08	67.7	91.2
	Dumbbell fly (pulley)	0.707	65.6	9.89	0.15	55.9	102.0
	Lat pulldown	0.593	139.1	13.74	0.09	126.5	193.3
	Single-arm row	0.711	97.5	14.58	0.15	56.9	112.8

## Data Availability

The data that support the findings of this study are available on reasonable request from the corresponding author. The data are not publicly available due to privacy and ethical restrictions.
